# Network control theory applied to the human connectome: A study on variability and discriminability of fMRI connectomic features under normal and defective sensorineural conditions

**DOI:** 10.1162/NETN.a.36

**Published:** 2025-11-20

**Authors:** Simone Papallo, Alessandro Pasquale De Rosa, Sara Ponticorvo, Mario Cirillo, Mario Sansone, Francesco Di Salle, Francesco Amato, Fabrizio Esposito

**Affiliations:** Department of Advanced Medical and Surgical Sciences, University of Campania “Luigi Vanvitelli,” Naples, Italy; Center for Magnetic Resonance Research, Department of Radiology, University of Minnesota, Minneapolis, MN, USA; Department of Electrical Engineering and Information Technology, University of Naples “Federico II,” Naples, Italy; Department of Medicine, Surgery and Dentistry, Scuola Medica Salernitana, University of Salerno, Salerno, Italy

**Keywords:** Connectome, Network control theory, Energy, Controllability, Sensorineural hearing loss

## Abstract

Network control theory (NCT) models human connectomes as high-dimensional input-state-output stable systems where the efficiency of neural connections can be addressed by energy cost (of state transitions) and controllability (from/to reachable states). Different options are available to extract NCT features: initial/final states, control time horizon, structural (vs. functional), and static (vs. dynamic) connectivity measure. Leveraging the minimum control paradigm, assuming the Schur stability for discrete systems, we investigate intra- and inter-individual variability of NCT features, across different settings and datasets, and assess their potential as useful connectome metrics in clinical studies. NCT was applied to structural and functional MRI (fMRI), in a cohort of 82 cognitively unimpaired elderly subjects with normal or (age-related) sensorineural condition (hearing loss), and in young adults from the Human Connectome Project database. Results demonstrated low intra-individual and moderate within-group inter-individual variability of NCT features. The energy cost was related to the time horizon of the system but did not discriminate groups. Controllability analyses revealed significant group effects and acceptable discrimination between normal and disease-affected connectomes, particularly for the default-mode network. We provide a systematic evaluation of different settings for fMRI-derived NCT features that may help guiding clinical applications toward capturing neurologically meaningful changes in the human connectome.

## Author Summary

Network control theory, modeling the human brain as a high-dimensional dynamic system, allows capturing the cascade of internal neural events at the base of spontaneous or stimulated brain fluctuations between cognitive states. The efficiency of neural connections can be addressed by energy cost (of state transitions) and controllability (from/to reachable states). Different options are available to derive energy cost efficiency and controllability for the human connectome using structural and functional MRI data. Starting from the mathematical foundations and leveraging the minimum control paradigm and the concept of Schur stability for discrete input-state-output systems, here, we show how to achieve stable estimates for energy cost and controllability using neural connectivity data in elderly subjects with normal or defective sensorineural conditions and in young subjects. We show that the energy cost depends on the control time horizon of the system, whereas controllability reveals significant and meaningful group effects, acceptably discriminating between normal and disease-affected connectomes. We provide a systematic evaluation of many different settings for energy cost and controllability which may help refining brain network control analyses, guiding clinical applications towards capturing neurologically meaningful changes, of the human connectome.

## INTRODUCTION

A convenient model for studying structural and functional connections across the entire human brain (the so-called connectome) should not only capture how well the underlying architecture facilitates the dynamic transitions between internal configurations (or functional states), supporting cognition in general, but also help revealing failures of sensorineural information processing under certain physiological (e.g., aging) or pathological conditions (Wang et al., 2022). Network control theory (NCT) (Karrer et al., 2020) might in principle help along such directions, albeit its applications to the study of human connectome are still limited, if compared to network graph theory (NGT) (Bullmore & Sporns, 2009).

Within the NCT framework, the human brain is depicted as a high-dimensional dynamic linear system that can be formally described by the so-called adjacency matrix, which algebraically aggregates all internal network connections to shape the connectome (Sporns, 2013). In general, when the system is assumed static (i.e., time-invariant), the adjacency matrix is obtained from structural connectivity (SC) neural data, for example, using brain tractography applied to diffusion MRI. Functional connectivity (FC) neural data can also be used to define the internal structure of the system (Honey et al., 2009), which will result as either time-invariant or time-variant, depending on whether the node interactions are estimated from the temporal correlations over the entire observational period (static FC [sFC]) or dynamically updated over shorter sliding time windows (dynamic FC [dFC]) (Hutchison et al., 2013). However, while NCT applications to the structural connectome were widely explored (Gu et al., 2015; Karrer et al., 2020; Parkes et al., 2024), the use of sFC or dFC in the context of NCT is not usual and less intuitive, considering mainly the peculiar nature of the adjacency matrix, which is based on the statistical dependency between (several) pairs of regional brain signals. Although the mechanism of information flow should imply the physical propagation of the signals along anatomical connections (Karrer et al., 2020), the FC level would also determine the actual capacity of the physical channel to transmit information (Has Silemek et al., 2024), according to the concept of “remote synchrony” (Vuksanović & Hövel, 2014). Particularly when considering the temporal evolution of dFC estimates, a variable level of synchronization would express the capability of the brain to switch toward states with distinct connectivity patterns and hub distributions, enabling a more (or less) flexible integration and propagation of information (J. Li, Zhang, et al., 2017). Thereby, if stimulating a certain region, the signal could be driven in a different region to an extent proportional to their baseline correlation. Along this line, the analysis presented by Deng et al. (2022) illustrated an NCT application that highlighted the possibility to extract NCT features to efficiently capture changes of the functional connectome across major large-scale networks of the human brain. Moreover, Deng et al. (2022) considered the use of functional MRI (fMRI) time series, not only to determine the multidimensional trajectories of the system across states but also to quantify the efficiency of state transitions in terms of control energy cost and system controllability, possibly as useful connectome features (Deng et al., 2022).

However, some methodological issues regarding the estimation of NCT features should be addressed. First, it is essential to implement appropriate normalization procedures for the adjacency matrix to prevent the generation of unstable systems, yet without affecting the accuracy of the model (Karrer et al., 2020). Second, the calculation of energy-related NCT features is affected by at least two choices for the model parameters (Stocker et al., 2023): (a) the control time, that is, the unitless control time horizon during which the system could reach a specific state (Deng et al., 2022) and (b) the selection of the initial and final states from the fMRI time series. Hence, this paper is designed to be illustrate the impact of these choices on the NCT features of the human connectome. Moreover, NCT is here applied using different combinations of SC and FC data from two groups of cognitively unimpaired aging subjects, one with normal hearing (NH) and one with age-related sensorineural hearing loss (HL) (Ponticorvo et al., 2022). In fact, this condition is not only acknowledged as a high-risk condition for later development of cognitive impairment (Irace et al., 2022) but is also a good pathological framework to test the discriminability performances of NCT features, in comparison with NGT features.

In this work, to help researchers focusing (or planning to focus) on brain network control applications to the study of age-related (or other disease-modified) defective conditions, we will first investigate the intra-individual variability of NCT features to determine the level of stability of these connectome features across initial and final conditions and different control times. We will apply different normalization approaches to derive energy and controllability features while ensuring the stability of the system. Second, the inter-individual variability will be also assessed to evaluate the stability of NCT features across three datasets. Ideally, a low intra-individual variability of the estimate features is desirable for the same individual FC profile, whereas a slightly higher inter-individual variability should be expected as the FC profile changes from subject to subject (Finn et al., 2015). Additionally, a novel energy-related feature is proposed to account for the static and dynamic properties of large-scale brain networks and the level of their inter-individual variability. We hypothesized that stable NCT features may also yield an acceptable discriminability power, possibly detecting disease-related alterations in the brain or even predicting clinical scores on an individual basis. As we principally analyzed a cohort of elderly subjects, we also included a separate cohort of healthy young adults from the public database of the Human Connectome Project (HCP) to verify the possibility of an age-related dependence of the observed stability for NCT features. Thereby, this work would not just (eventually) corroborate the results presented in Deng et al. (2022) but it would also investigate the variability of NCT features across normal and diseased individuals and across different age ranges, exploring their discriminability between normal and pathological subjects to illustrate the potential of the methodology in obtaining valid candidate biomarkers of the human connectome.

## MATERIALS AND METHODS

### Subjects and Datasets

All details about subjects, including their clinical assessment, and the detailed procedures for MRI data acquisition, preprocessing, and matrix generation can be found in Ponticorvo et al. (2022). Individual de-identified participant raw imaging data in NIfTI (Neuroimaging Informatics Technology Initiative) format are available on OpenNeuro (see https://openneuro.org/datasets/ds005026/versions/1.0.0).

Briefly, structural and functional neural connectivity data were obtained from a total of 82 human subjects, of which 52 (36 males) aged 63.7 ± 7.8 (mean ± standard deviation) with sensorineural HL and 30 (10 males) aged 59.5 ± 7.2 years with NH. All subjects were free from cognitive impairment based on the age- and education-adjusted Italian Montreal Cognitive Assessment (Santangelo et al., 2015). Pure-tone average (PTA) and speech reception threshold (SRT) in HL subjects were respectively 50.3 ± 15.1 dB and 40.9 ± 22.1 dB (mean ± standard deviation) for the right ear and 50.3 ± 13.1 dB and 37.1 ± 22.2 dB (mean ± standard deviation) for the left ear.

Diffusion and blood oxygen level dependent (BOLD) fMRI images were registered to 3D T1-weighted MRI images, which were in turn normalized to the MNI (Montreal Neurological Institute) template to provide the transformations necessary to apply the brain parcellation to structural and functional data in the native space. Two hundred bilateral cortical nodes were derived from the functional local–global normative parcellation (Schaefer et al., 2018) and accordingly categorized into set of nodes corresponding to seven large-scale canonical functional networks: visual network (VIS), somato-motor network (SMN), dorsal attention network, salience ventral attention network, limbic network, fronto-parietal network, and default mode network (DMN) (Thomas Yeo et al., 2011). The brain parcellation applied to the data resulted in a 200 (node/regions) × 200 (node/regions) symmetric SC matrix (as obtained from whole-brain tractography) and a 200 (node/regions) × 400 (time points) functional time-course matrix (as obtained by averaging the BOLD fMRI time series across all voxels in the corresponding atlas region), per subject.

An additional dataset was included in the analysis. Particularly, we selected 30 healthy subjects (see Supporting Information for IDs of HCP subjects), numerically matched with our NH subjects, (20 females with age range of 22–35 years) from the HCP Young Adult dataset 1200 Subjects Release and applying the same parcellation, extracting 200 × 1,200 (Region × TR) time series per subject (see https://db.humanconnectome.org/data/projects/HCP_1200 for detailed procedures of MRI data acquisition and preprocessing).

### NCT Model and Features

The following dynamic linear system model is commonly used to derive NCT features using neural connectivity data:

$$\dot{x\;}=A\left(t\right)∙x\left(t\right)+B∙u\left(t\right)$$ (1)

Here, x(t) is an N × 1 vector representing the system (brain) state at time *t* (N = number of nodes), A(t) is called adjacency matrix and is an N × N symmetric matrix (with zero diagonal entries) representing the interactions between each pair of nodes at time *t*, B is called the input control matrix and is an N × K matrix, K being the dimensionality of a K × 1 input energy signal u(t) controlling the system along a state trajectory from a given initial state x(0) = x_0_ to a given final state x(T) = x_T_.

The minimum energy control principle applied to the system (a) makes the trajectory of the system physically plausible for any pairs of initial and final states but requires a proper normalization of the adjacency matrix using its weighted Laplacian matrix L = {L_ij_} (Tang & Bassett, 2018).

Thus, the following normalization procedure is applied to matrix A = {A_ij_}:

$$L_{ij}=\delta_{ij}\sum_{k}\left|A_{ik}\right|-A_{ij}$$ (2)

$$A\leftarrow \frac{-L}{\lambda_{\max}\left(L\right)},$$ (3)

where *δ*_ij_ = 1 if i = j and *δ*_ij_ = 0 if i ≠ j, and *λ*_max_ is the maximum absolute eigenvalue of L. This normalization ensures that the eigenvalues of the adjacency matrix are negative and ranging between −1 and 0, a condition sufficient for stability and reachability (Datta, 2004).

Based on these assumptions, for a given initial state x_0_ and a given final state x_f_, and further assuming the matrix A does not vary across time (time-invariant system), the minimum control energy principle allows us to calculate a static energy cost (E_s_) of each transition as:

$$E_{s}=\frac{1}{2}d^{T}W^{-1}d$$ (4)

where:

$$d=x_{f}-e^{A*\tau }x_{0}$$ (5)

Here, W is the so-called controllability Gramian matrix of the system (Tang & Bassett, 2018) and *τ* is the control time, which represents the unitless control time horizon of the system, that is, the evolution time of the system to reach the final state within a given tolerance when all transient effects have decayed, or, in other words, the time units during which the decay of the input energy occurred (Deng et al., 2022).

Assuming that the system is being controlled via one single node (i) at time, that is, B = B_i_ = diag(0, …, 1, …0) with 1 only in the *i*-th diagonal entry, the controllability Gramian matrix can be either obtained as the solution of the Lyapunov equation for the system in Equation (1) (Poznyak, 2008) or numerically by using the more general integral formulation:

$$W_{i}=\int_{0}^{\infty }e^{At}B_{i}B_{i}^{T}e^{A^{T}t}dt$$ (6)

$$AW_{i}+W_{i}A^{T}+B_{i}B_{i}^{T}=0$$ (7)

While less computationally efficient, the formula in Equation (6) is chosen as an alternative to Lyapunov solution when the maximum absolute eigenvalue of the adjacency matrix is too close to zero with respect to the numeric tolerance.

While this double formulation is acceptable for estimating the energy cost, to make the extraction of controllability features more feasible and meaningful, here, we used a slightly different normalization for the adjacency matrix, ensuring that all absolute eigenvalues of the discrete-time version of the system are strictly ranging between 0 and 1 (so-called Schur stability [Karrer et al., 2020])

$$A\left(t\right)\leftarrow \frac{A}{1+\lambda_{\max}\left(A\right)}$$ (8)

Particularly, this normalization allows us to use simplified expressions for the so-called average controllability (AC) and modal controllability (MC) (Wang et al., 2022) to quantify the ability to drive the system from a single node respectively toward more “consolidated” states and toward more “difficult-to-reach” states. A consolidated state would be a state that is well-established from previous recurrent experiences and therefore expected or found to occur naturally and frequently during a given period of observation. In contrast, a nonconsolidated or difficult-to-reach state would be one that can be reached upon very specific conditions of the brain (e.g., via certain task demands to the subject) and therefore expected to occur less frequently during a given period of free observation (e.g., with the subject at rest).

AC and MC can be evaluated as:

$${AC}_{i}=\sum_{j=1}^{N}\frac{\upsilon_{ij}^{2}}{\left(1-\lambda_{j}^{2}\left(A\right)\right)}$$ (9)

$${MC}_{i}=\sum_{j=1}^{N}\left(1-\lambda_{j}^{2}\left(A\right)\right)\upsilon_{ij}^{2}$$ (10)

All the above formulations are strictly valid when the adjacency matrix is constant (i.e., the system is time invariant) for the entire time of dynamic observation. However, in order to handle the more general case of variable *A*(*t*), we use the approximation of discrete dynamic temporal networks (A. Li, Cornelius, et al., 2017) by dividing the time window of observation into *M* shorter time windows. For each of short window, the adjacency matrix is assumed constant:

$$A\left(t\right)=A_{m}\mathrm{with}m=1\ldots \ldots ..M$$ (11)

Thus, under the same minimum control energy constrain, we define the dynamic energy (*E*_*d*_) as:

$$E_{d}\left(x_{0},x_{f}\right)=\frac{1}{2}d^{T}W_{dyn}^{-1}d$$ (12)

$$d=x_{f}-e^{A_{M}\tau_{M}}e^{A_{M-1}\tau_{M-1}}\ldots e^{A_{1}\tau_{1}}x_{0}$$ (13)

With the controllability Gramian matrix given by:

$$W_{dyn}=SWS^{T}$$ (14)

$$S=\left(\prod_{i=M}^{2}e^{A_{i}\tau_{i}^{d}},\ldots ,\prod_{i=M}^{m+1}e^{A_{i}\tau_{i}^{d}},I\right)$$ (15)

$$W=\text{diag}\left(W1,W2,\ldots .WM\right)$$ (16)

### NCT Application to Connectome Data

In (1), each scalar component of the vector x(t) describes the activation state of one region (node) at time t and the order of scalar elements (*N* = 200 in our case) corresponds to the order of regions in the chosen brain parcellation. The signal describing this component can only be filled using regional fMRI time series at the observed time points. When simulating the system for extracting NCT features, *A*(*t*) can be estimated from either the structural or the FC matrix.

In the former case the adjacency matrix *A*(*t*) = A_S_ is assumed to be constant over time and the system is time invariant. The entries of A_S_ are set from the symmetric SC matrix obtained via whole-brain tractography (see Ponticorvo et al., 2022, for more details). In the latter case, *A*(*t*) can be assumed to be either constant, that is, *A*(*t*) = *AF* and the system is time invariant, or variable over time, that is, *A*(*t*) = A_F_(*t*), and the system is time variant.

For the time-variant case, a shorter time window (*t* − *δ*, *t* + *δ*) is used to determine A_F_(*t*). Hence, the adjacency matrix is allowed to change over time and its updates over time result from using a (sliding) time window approach to estimate the so-called dynamic FC (Leonardi & Van De Ville, 2015). If the time window is considered of infinite duration (i.e., *δ* → ∞), the elements of matrix A_F_ are estimated from the so-called sFC, that is, from the temporal correlations over all available time points in the window of observation. Therefore, A_F_ is kept constant during any simulated trajectory of the state vector. In this work, the static and dynamic FC are calculated as the Pearson correlation matrix between all pairs of regional fMRI time courses. For the dynamic FC, we divide the signal into M windows and then calculate the Pearson correlation for each window. Thus, matrix A is always symmetric and with values ranging from −1 and 1. In this matrix, diagonal entries are always set to zero, whereas we considered two separate cases for the negative entries, which were either kept at their original value or set to zero (thus making A positive semidefinite). In fact, while some fMRI studies separate positive and negative entries in the FC matrix, focusing on the positive-definite FC matrix to reduce the impact of spurious anticorrelations in the fMRI signals after preprocessing (see, e.g., van den Heuvel et al., 2017), other fMRI studies have shown that negative connections can contribute to the controllability of the system (see, e.g., Manjunatha et al., 2024).

Matrix B is used to account for controlled dynamics in the model. Setting the element of matrix B corresponds to select or weight K input energy signals in u(t) across all N nodes. B is kept constant in our model and shaped in such a way that control nodes can be selected independently from the input energy signals. For example, it can be set in such a way to simulate the evolution of the state vector when only one specific node is selected to drive the system toward the desired state (B = Bi). The input signal can in principle be thought as forced via an external stimulus or intrinsically generated from the spontaneous activation of an internal cognitive control process aiming at driving the system from one activation state to another activation state. In NCT, matrix B serves the purpose to describe the role of the nodes through which the brain is induced to deviate from an ongoing trajectory by the injection of energy via internal or external pathways. Given the linearity of the model, assuming the application of the input energy signal at each single node, one at time, does not impair the ability to characterize the system controllability (at that node). Therefore, here, we calculated NCT features across all nodes belonging to the same large-scale network. Thereby, we have set to 1 the elements of matrix B corresponding to the selected nodes and consequently a single NCT feature for each large-scale network was extracted.

### NCT Feature Analysis

NCT features were estimated from neural connectivity data and analyzed for their variability within subject by considering two different age range data sets and for both variability across, and discriminability between, NH and HL groups.

The NCT feature estimation was performed in MATLAB (The Mathworks, Inc., www.mathworks.com) by looping blocks of instructions taken from the original code shared by Deng et al. (2022; https://github.com/brain-intelligence-lab/functional_controllability). Energy-related features were derived by repeatedly performing simulations with randomly selected initial and final states and combining different types of time-invariant and time-variant estimation of the adjacency matrix. Similarly, controllability features were derived from all types of estimation of the adjacency matrix. More specifically, five different scenarios were considered: (a) time-invariant interaction from SC, (b) time-invariant interaction from sFC, (c) time-invariant interaction from static functional connectivity with negative values set to zero, (d) time-variant interaction from dFC, and (e) time-variant interaction from dynamic functional connectivity with negative values set to zero. Two simulations were performed. In the first case, we have selected 20 pairs of initial and final states by randomly extracting two time points from the first 25% and the last 25% time points of the fMRI time courses to define the initial and final activation states, respectively. According to this selection scheme, we extracted plausible initial states and effectively reachable final states, because we selected these states in chronological order directly from the fMRI sequence, keeping the natural sequence of states of the subject at the rest. We estimated the energy cost necessary to steer the brain from the initial to the final state by varying the control time horizon of system in a discrete range from 10 to 400 with a step of 20 units. Moreover, for the time-variant definition of the adjacency matrix, the size of the sliding time window was also varied and set to 30 s, 50 s, and 100 s. In the second case, we repeated the same selection scheme for one pair of initial and final states from the fMRI sequence and the energy-related feature was estimated by letting the control time horizon of the system to vary in the same discrete range and by considering a window size of 50 s for the dynamic case. After inspecting the trends in the control energy cost for all types of interaction and for different time window size, we selected four specific control times and one specific time window size for further assessment. Here, we considered the logarithm of the energy values to ensure the normality of the data distribution and improve the scale of the graphs. Moreover, as we expected differences emerging between time-variant (dynamic) and time-invariant (static) cases as being indicative of the dynamic energy efficiency of the system (Deng et al., 2022), we considered the ratio of the two log-E values as a potentially interesting energy-related NCT feature.

However, the intra- and inter-individual variability of NCT features were assessed via the coefficient of variation (CoV) to quantify numerically the impact of the model parameters (or group membership) on the stability of the features. A comparable inter-individual variability could provide an indication for the robustness of the features, ensuring the possibility to define potential biomarkers for brain (dys)functioning, when comparing differently age ranged or healthy versus impaired subjects.

Specifically, the stability of this energy feature across repeated simulations was preliminary assessed on five (randomly selected) NH subjects. Namely, the energy cost was repeatedly assessed for each random NH subject 20 times (one for each control time) for 20 different pairs of random initial and final states and for the three values set for the time window size. This preliminary analysis allowed us to assess the stability of the energy cost estimation from simulations with respect to a different (random) selection of the initial and final states and to verify the impact of the time window size on the intra-individual CoV, which was found sufficiently stable (i.e., lower than 5%). We reported the mean value of the intra-individual CoV across the five NH subjects. Thus, subsequently, referring to this energy-related NCT feature, we proceeded with assessing the inter-individual CoV by repeating simulations in all subjects for a single pair of randomly selected initial and final states and for four control times (*τ*_s_ = 30, *τ*_s_ = 50, *τ*_s_ = 100, *τ*_s_ = 200). In the inter-individual analysis, we consider three dynamic versus static energy ratios as candidate energy-related NCT features for control dynamic energy efficiency of the system: (a) the ratio between energy cost values when considering the sFC and dFC as adjacency matrix, (b) the ratio between energy cost values when considering the sFC and dFC as adjacency matrix but setting negative values to zero, and (c) the ratio between energy cost values when considering SC and dFC as adjacency matrix. The AC and MC were also derived for the five types of adjacency matrix described above after the more appropriate normalization had been applied to ensure the stability of the discrete-time system. To assess the variability and discriminability of all the above features, we calculated the mean value and inter-individual CoV for each experimental group. The effect of the group (NH vs. HL), the linear discrimination, and the correlations with clinical scores (PTA, SRT) were calculated as follows: The effect of the group was obtained via a linear regression model describing the feature as a function of age, sex, and group membership. The mean values for the two groups and the *p* value for the group coefficient is reported. The linear discrimination was instead performed via receiver operating characteristic (ROC) analysis and here reported as area under the curve (AUC). Spearman *ρ* correlation was used to calculate the correlation between NCT feature and clinical scores for each ear (PTA, SRT). Results were considered descriptively significant when *p* value was lower than 5%. Moreover, to assess the impact of NGT features on NCT features, the previous analysis was also performed by adding the estimates of two major NGT features, that is, network strength and density, as covariates for the controllability analysis. The results were again presented in terms of *p*-value for the group coefficient via linear regression. False discovery rate (FDR) correction was applied to account for multiple comparisons across different canonical networks (Benjamini & Yekutieli, 2001).

## RESULTS

All the results from the analyses on the variability and discriminability of NCT features are reported in the following subsections. In all cases, NCT features were calculated using all nodes belonging to the same large-scale network as control nodes.

### Intra-Individual Variability of Energy Cost

Starting from Equations (4) and (12), the logarithmic values of the energy cost, when a time-variant and time-invariant system was respectively considered, were calculated. To assess the variability of the energy cost with respect to the different initial and final states in each simulation, we computed the intra-individual CoV across all (20) choices of the initial and final random states, separately for five (randomly selected) NH subjects and four specific control times (*τ*_s_ = 30, *τ*_s_ = 50, *τ*_s_ = 100, *τ*_s_ = 200). The mean CoV across all subjects is reported for the DMN in Table 1.

**Table 1.** Estimated mean CoV across the five random NH subjects for the energy cost values for the DMN across five different definitions of the adjacency matrix

a) Energy case Windows size: 30 s	CoV [%] (*τ*_s_ = 30)	CoV [%] (*τ*_s_ = 50)	CoV [%] (*τ*_s_ = 100)	CoV [%] (*τ*_s_ = 200)
sFC 30 s	2.04	2.04	2.04	2.04
sFC no neg values 30 s	2.39	2.39	2.39	2.39
dFC 30 s	2.43	3.25	3.75	3.69
dFC no neg values 30 s	2.96	4.58	6.47	6.12
Structural matrix 30 s	2.80	2.83	2.84	2.84

b) Energy case Windows size: 50 s	CoV [%] (*>τ*_s_ = 30)	CoV [%] (*τ*_s_ = 50)	CoV [%] (*τ*_s_ = 100)	CoV [%] (*τ*_s_ = 200)
sFC 50 s	2.05	2.05	2.05	2.05
sFC no neg values 50 s	2.51	2.51	2.51	2.51
dFC 50 s	3.26	3.07	3.31	2.93
dFC no neg values 50 s	4.45	5.54	5.87	5.56
Structural matrix 50 s	3.33	3.37	3.39	3.39

c) Energy case Windows size: 100 s	CoV [%] (*τ*_s_ = 30)	CoV [%] (*τ*_s_ = 50)	CoV [%] (*τ*_s_ = 100)	CoV [%] (*τ*_s_ = 200)
sFC 100 s	2.03	2.03	2.03	2.03
sFC no neg values 100 s	2.63	2.63	2.63	2.63
dFC 100 s	2.64	2.85	2.85	2.85
d-FC no neg values 100 s	3.65	3.72	3.57	3.57
Structural matrix 100 s	3.05	3.07	3.08	3.08

The simulations were repeated for 20 pairs of initial and final states and four different control times (*τ*_s_ = 30, *τ*_s_ = 50, *τ*_s_ = 100, *τ*_s_ = 200). (a) The window size is set to 30 s. (b) The window size is set to 50 s. (c) The window size is set to 100 s. For all window sizes and independently of the adjacency matrix definition, a low CoV (<5%) across the four control times here suggests that the selection of the starting point weakly affects the estimate of the energy cost.

Independently of the adjacency matrix (and the window size), for the DMN, as well as for the SMN and VIS networks (as shown in the Supporting Information), the intra-individual variability analysis of the energy cost resulted in a mean CoV < 5%, suggesting that the selection of the starting point weakly affects the estimate of the energy cost. Moreover, the mean intra-individual CoV appears to be stable with respect to the control time.

**Figure 1.**
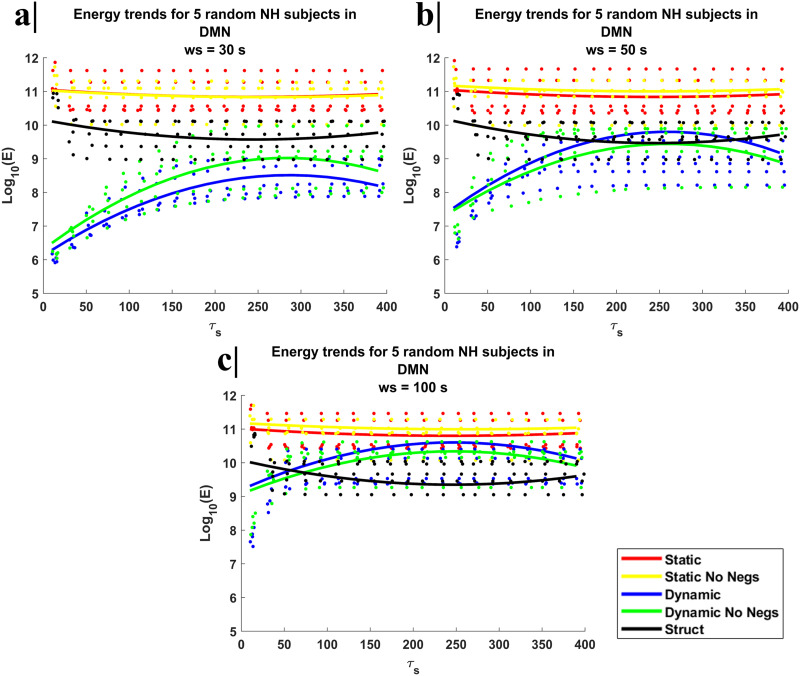
The plots represent the mean energy values per subject across the 20 pairs of initial and final time points for five random NH subjects and the trend of mean energy cost values across the subjects for the DMN as a function of control time (*τ*_s_) and for five different scenarios to define adjacency matrix and for three different window size: (a) window size set to 30 s, (b) window size set to 50 s, and (c) window size set to 100 s. In all plots, we report the energy cost trends respectively when the adjacency matrix is identified as sFC, that is, the static functional connectivity matrix with negative values (red) and with negative values set to zero (yellow), as dFC, that is, the dynamic functional connectivity with negative values (blue) and with negative values set to zero (green) and as SC, that is, the SC matrix (black). For all window sizes and lower control times (<50), the energy cost associated with the trajectory of the system from the initial to the final state is significantly lower when the adjacency matrix is dynamically updated over time compared to the static cases.

Furthermore, in Figure 1, we reported the trends in the energy cost across different control times for the DMN, that is, the main large-scale network connecting posterior, anterior, and lateral regions of the brain. For all window sizes and lower control times (<50), we noted that the energy cost associated with the trajectory of the system from the initial to the final state is significantly lower when the adjacency matrix is dynamically updated over time (i.e., time-variant system with dFC data) compared to the static cases (i.e., time-invariant system with SC or sFC data). However, the window size expectedly affected such trends. Specifically, the shorter the window size the larger the maximum control time for which the dynamic update of the interaction matrix results in a reduction of the energy cost compared to the static case. Indeed, for a window size of 30 s, and control times up to over 150, the dynamic adjacency matrix (either with negative values set to zero or not) always results in a lower energy cost compared to the static cases (either with negative values set to zero or not), whereas, for a window size of 100 s, this energetic advantage can be dismissed. However, the window size also affected the energy cost in case of time-invariant system (with static functional connectivity) because the window size also determines the selection of the final point that should be reached by the system. Across all three window sizes, the energy cost does not vary noticeably with control time when the adjacency matrix is based on sFC, whereas a negative trend, more prominent for the longest window size, is observed for the time-invariant system with adjacency matrix defined from SC, as lower values for the energy cost occur at higher control times. In contrast, the energy cost for the dynamic cases always increases from lower to intermediate control times.

The intra-individual variability analysis was repeated on the HCP dataset confirming the previous consideration about energy trends, where the energy cost is still significantly lower when the adjacency matrix is dynamically updated over the time compared to the static case, and about the low mean CoV (<5%) across the three window sizes and the four control time (see Supporting Information).

### Inter-Individual Variability of Energy Cost

To assess the variability of the energy cost across a whole sample of subjects, we randomly selected a single pair of initial and final states, choosing the same time points for all subjects, from the initial and final part of the functional time series to perform a trajectory simulation and reported energy cost trends for each group (NH and HL) separately at an intermediate value for the window size (50 s). Then, we evaluated the inter-individual CoV for each group separately, when considering four control times (*τ*_s_ = 30, *τ*_s_ = 50, *τ*_s_ = 100, *τ*_s_ = 200) in the DMN and in two more resting-state sensory networks (VIS and SMN), which could be also expected as relevant for the present application given the sensorineural deficit of HL subjects. Independently of the control time and the group membership, the inter-individual CoV ranged between 5% and 10%, except for the SMN, where it was marginally higher than 10%. The inter-individual CoV for the HCP dataset confirmed this variability increment (see Supporting Information for tables and figures about HCP subjects results).

**Figure 2.**
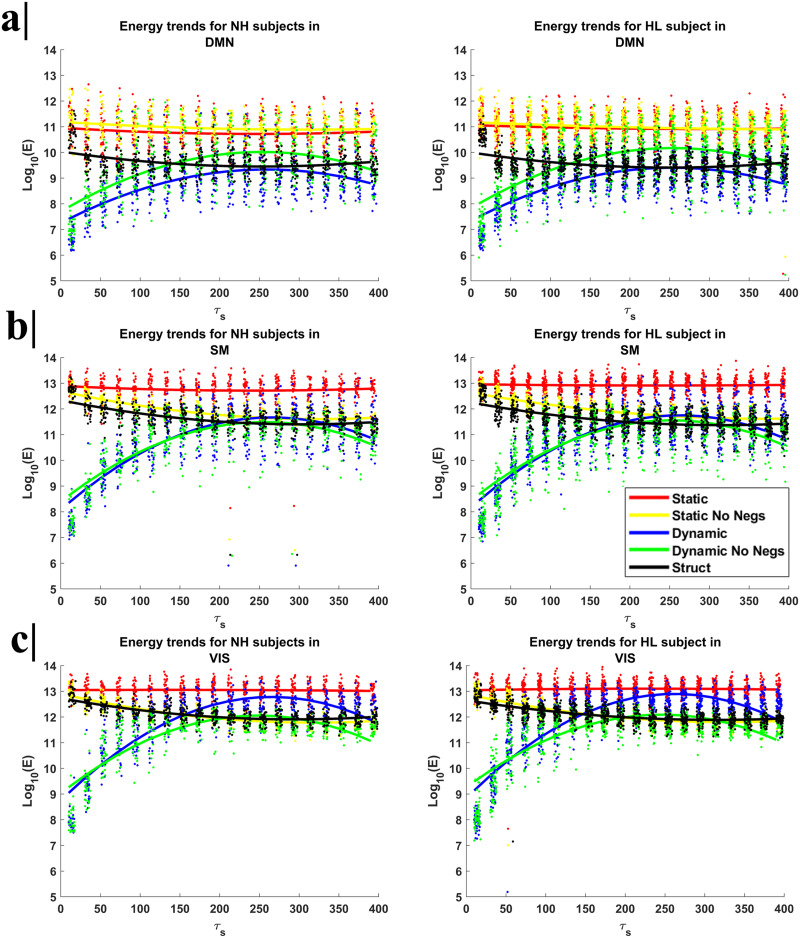
The plots represent the energy value of each subject at each control time and the trend of mean energy cost values across subjects for an intermediate window size (50 s) as a function of control time (*τ*_s_), respectively (a) for the DMN, (b) for the SMN, and (c) for the VIS, and separately for NH (on the right) and for HL (on the left) subjects. The energy cost trends are reported respectively for an adjacency matrix defined from sFC, that is, the static functional connectivity matrix with negative values (red) and without negative values (i.e., set to zero) (yellow), from dFC, that is, the dynamic functional connectivity with negative values (blue) and without negative values (green) and as SC, that is, the SC matrix (black). Even at the group level, it is possible to observe the reduction of the energy cost for shorter control time (<50), when the system is identified by a time-variant (dynamic) adjacency matrix.

In addition, in Figure 2, we displayed the energy cost trends at the group level (HL and NH groups) for the three selected resting-state networks (DMN, VIS, and SMN). Even at the group level, we observed the reduction of the energy cost for shorter control time (<50), when the system was identified by a time-variant (dynamic) adjacency matrix. For the DMN, the energy cost was in good approximation constant with respect to the control time when the system was time invariant and identified via the sFC (whether with negative values were set to zero or not). However, for the SMN and VIS networks, setting sFC negative values to zero determined a more rapid reduction of the energy cost at higher control times, down to levels comparable with those reached by the system when the adjacency matrix is dynamically updated over time. The same trends are observed for both NH and HL subjects. Comparing the energy trends from the two groups at each control time (two-sample *t* test), after FDR correction, the sFC energy trend for NH was significantly lower than corresponding energy trend of HL for higher control time (>100) in the DMN (minimum *T*(80) = 2.30, *p* = 0.0241) and SMN (minimum *T*(80) = 3.20, *p* = 0.0020), whereas no significant differences were detected for the VIS (*p* > 0.05).

### Discriminability Power of Dynamic Energy Efficiency

Here, we referred to the ratio of Log-E values between static (time-invariant) and dynamic (time-variant) conditions as a candidate energy-related NCT feature of dynamic energy efficiency, as illustrated in Paragraph 2.4.

**Figure 3.**
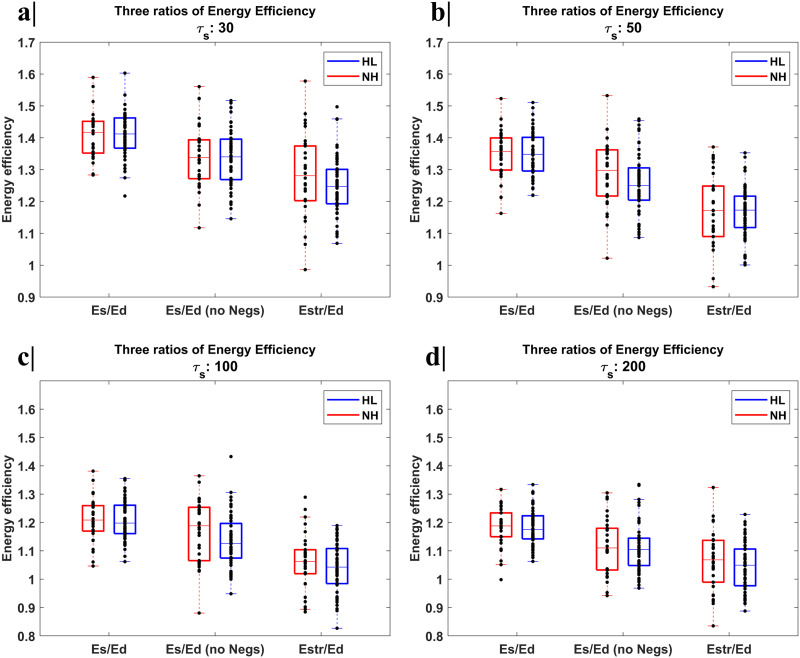
Boxplots with the distribution of the energy-related NCT feature values for the DMN, when considering three different energy ratios to describe the dynamic energy efficiency of the system, separately for each group (NH in red and HL in blue) and for four control times: (a) control time set to 30, (b) control time set to 50, (c) control time set to 100, and (d) control time set to 200. For all control times and all definition of the dynamic energy efficiency, the inter-individual CoV, the *p* value of the group variable from the linear regression (after FDR correction) and the AUC-ROC were estimated. These energy-related feature exhibits a moderate inter-individual variability with CoVs ranging between 5% and 10% in both NH and HL groups, with no significant differences (*p* > 0.05) and no acceptable discriminability (AUC-ROC < 0.7) between the two groups.

For all control times, this energy-related feature exhibited an inter-individual CoV ranging between 5% and 10% in both NH and HL groups, with no significant differences (*p* > 0.05) and no acceptable discriminability (AUC-ROC < 0.7) between the two groups. Negative correlations between the dynamic energy efficiency, when considering the sFC and dFC with negative values set to 0 and a control time set to 200, and PTA of both ears (*ρ* right ear = −0.373 and *p* value = 0.010 and *ρ* left ear = −0.352 and *p* value = 0.015) and positive correlation between the dynamic energy efficiency, when considering sFC and dFC for the adjacency matrix and the control time set to 200, and the SRT of the left ear (*ρ* left ear = 0.291 and *p* value = 0.047) were found respectively for the DMN and VIS networks. Albeit correlations between dynamic energy efficiency metrics and the clinical scores emerged, none of them survived multiple comparison FDR correction. In Figure 3, we reported the boxplots for the three different measures of dynamic energy efficiency for the DMN separately for each group and for four control times.

### Variability and Discriminability Power of Controllability Features

**Figure 4.**
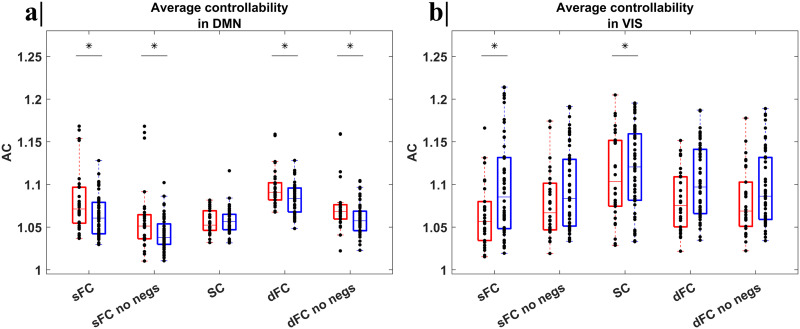
Boxplots with the distribution of the AC values in the (a) DMN and (b) VIS networks, separately for each group (NH in red, HL in blue), considering five different scenarios to define the adjacency matrix (respectively the sFC, sFC with negative values set to zero, SC, dFC, and dFC with negative values set to zero). The inter-individual CoV, *p* value of group variable via linear regression, and the AUC-ROC were calculated. Significant differences (*p* < 0.05, *) between the two groups, after FDR correction, were highlighted. For the DMN (a), we noted a significant increase of the AC values in the NH, compared to the HL subjects, when the sFC, with and without negative entries (respectively, *p* = 0.011 and *p* = 0.019) and dFC, with and without negative entries (respectively, *p* = 0.013 and *p* = 0.038), were considered as adjacency matrix. For the VIS (b), a significant increment in the AC values emerged for the HL, compared to NH, subjects, when the sFC and the SC (respectively, *p* = 0.016 and *p* = 0.035) were considered as adjacency matrix. For all cases, a lower inter-individual variability, with CoV around or below 5%, was found. In no case, a significant difference is associated with an acceptably discriminate between groups (AUC < 0.7).

**Figure 5.**
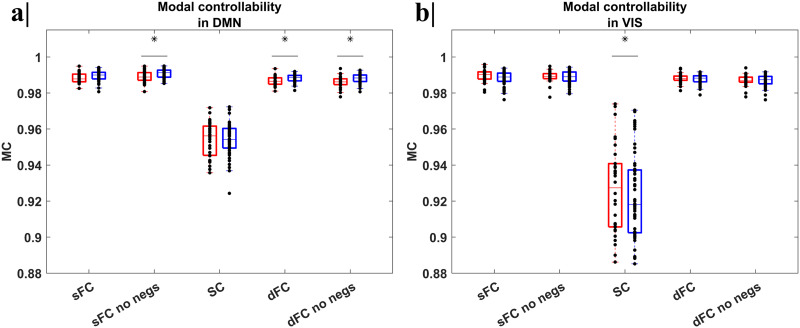
Boxplots with the distribution of the MC values in the (a) DMN and (b) VIS networks, separately for each group, considering five different scenarios to define the adjacency matrix (respectively the sFC, sFC with negative values set to zero, SC, dFC, and dFC with negative values set to zero). The inter-individual CoV, *p* value of group variable via linear regression, and the AUC-ROC were calculated. Significant differences (*p* < 0.05, *) between the two groups, after FDR correction, were highlighted. For all cases, a remarkable lower inter-individual variability, with CoV around 1%, was found. For the DMN (a), we noted a significant increase of the MC values in the NHs, compared to the HLs, when the static sFC without negative entries (*p* = 0.013), and dFC, with and without negative entries, were considered as adjacency matrix (respectively, *p* = 0.022 and *p* = 0.013) with an acceptably discrimination power between groups (respectively, AUC = 0.706 and AUC = 0.712) as well. For the VIS (b), a significant increase of the MC emerged in the NH, compared to the HL subjects, when the SC (*p* = 0.030) was considered as adjacency matrix. No acceptable discriminability (AUC > 0.7) was found.

Starting from the Equations (9) and (10), mean values of AC and MC were estimated for each subject and then averaged for all seven networks together, across the two groups, reporting the *p* value of the group effect (from linear regression) and the discrimination power (AUC) from the ROC analysis. The inter-individual CoVs were also calculated. In Figures 4 and 5, we respectively reported the AC and MC values distributions in the (a) DMN and (b) VIS networks, separately for each group (NH in red, HL in blue), considering five different scenarios to define the adjacency matrix for each adjacency matrix and separately for each group. The boxplots for the SMN are reported as well in the Supporting Information.

The AC showed a reduced inter-individual variability than energy-related features with a CoV ranging from ∼1% to ∼5% for NH and HL groups, while a CoV ranging from ∼4% to ∼7% was found for the HCP subjects. Overall, the dynamic AC (when considering the dFC as adjacency matrix) was on average higher than static AC (when considering alternatively the SC or sFC as adjacency matrix) for both datasets.

Significant differences between the means of the two groups (*p* < 0.05) for both the DMN and VIS networks were found. Specifically, for the DMN, both the static and dynamic AC were significantly higher in NH, compared to HL, subjects, when defining the adjacency matrix based on the sFC (*p* = 0.011) and dFC either with or without negative values (respectively, *p* = 0.013 and *p* = 0.038). However, in no case this feature acceptably discriminates between groups (AUC < 0.7). Instead, for the VIS, only the static AC, when the time-invariant adjacency matrix is defined via sFC (with negative values) or SC, was significantly higher in HL, compared to NH, subjects, but, anyway, this feature did not still acceptably discriminate between groups (AUC < 0.7). In terms of clinical correlations, the structural AC of the DMN was positively correlated with the PTA of the right ear (*ρ*_right ear_ = 0.299 and *p* value = 0.041) whereas the static AC of the SN was positively correlated with the SRT of the right ear (*ρ*_right ear_ = 0.304 and *p* value = 0.038).

The MC showed a remarkably reduced inter-individual variability with a CoV below ∼1% for both groups, as also confirmed for the HCP datasets. Overall, static MC, when considering sFC either with negative values or not as adjacency matrix, is on average higher than dynamic MC, when considering the dFC either with negative values or not as adjacency matrix, for both groups. Significant differences between the means of the two groups (*p* < 0.05) were found in DMN and VIS, but only acceptably discriminating in the DMN.

Specifically, for the DMN, both the static and dynamic MC were significantly higher in HL, compared to NH, subjects, when defining the adjacency matrix based on the static without negative entries (*p* = 0.013) and dynamic functional connectivity with and without negative values (respectively, *p* = 0.022 and *p* = 0.013). However, the dynamic MC as defined on the dFC (with or without negative values) acceptably discriminated between groups (respectively, AUC = 0.706 and AUC = 0.712). No significant difference when the time-invariant adjacency matrix was based on the SC. Instead, for the VIS, only the static MC, when the time-invariant adjacency matrix was defined based on SC, was significantly higher in HL, compared to NH, subjects, albeit this feature did not acceptably discriminate between groups (AUC < 0.7). However, the static MC of the VIS network was positively correlated to the SRT of the right ear (*ρ*_right ear_ = 0.392 and *p* value = 0.007). By contrast, the analysis of NGT features showed significant differences only for the VIS network (*p* < 0.05) and not for the DMN, independently of the selected adjacency matrix. However, the analysis of NCT features where two major NGT features, that is, network strength and density, were included as covariates did not show any statistical differences between the two groups (see Supporting Information).

## DISCUSSION

NCT may represent a new frontier in the study of the human connectome, albeit with some outstanding applications already proposed (Medaglia et al., 2017; Papallo et al., 2024; Scheid et al., 2021; Wang et al., 2022). Although the use of SC is far more consolidated in the context of NCT (Gu et al., 2015; Karrer et al., 2020; Parkes et al., 2024), in the recent years, the use of FC data has also assumed a great relevance thanks to the capability of dynamic FC to efficiently capture the brain dynamics of large-scale brain networks (Deng et al., 2022). A previous work had already suggested how both sFC and dFC data could be beneficially employed to predict individual scores in behavioral tasks (Liégeois et al., 2019). Here, we explored different combinations of SC and FC data from two experimental groups of NH and HL aging individuals (Ponticorvo et al., 2022), and from a public dataset of healthy subjects from the HCP spanning a different age range, to test the impact of several methodological choices and to assess the current potential for a more clinical NCT application to the human connectome.

First, our work replicated, across different experimental datasets, the results shown by Deng et al. (2022), particularly confirming the advantage, in terms of energy costs, of using the dynamic (functional), rather than static (functional or structural) connectivity, with an adjacency matrix updated over time. In other words, the human connectome “seen” as a time-variant dynamic system may effectively evolve by constantly adapting the structure to the trajectory to reduce the overall energy cost of the transitions (Hutchison et al., 2013). However, only when SC data are used, there seems to be a specific control time for which no differences are evident between static and dynamic conditions. This is in full agreement with the results from Deng et al. (2022), where it is outlined how dFC retraces the underlying SC for control times above a certain value. Second, more detailed methodological considerations are here provided with respect to the different settings of a similar NCT analysis. Namely, we selected the appropriate matrix normalization procedure, ensuring the Schur stability of the model according to Karrer et al. (2020), and assessed both the intra-individual variability of the resulting NCT features across different control times and window sizes and the inter-individual variability of the NCT features across different experimental groups, particularly examining their discriminability power in the comparison between normal and sensory-impaired conditions.

### Methodological Considerations

When the system was simultaneously driven via the nodes of the main large-scale network, that is, the DMN, the intra-individual NCT analysis showed that energy cost trends were sufficiently stable (CoV < 5%) across different combinations of model settings, suggesting a good capability of the NCT model to outline the overall cost of state transitions even when the initial and final states were randomly selected. Nevertheless, we have also showed that the window size may still affect the estimation of the energy cost, even when assuming a time-invariant system, because while the adjacency matrix does not change over time, the duration of the time window is still involved in the selection of the final state. We also sought to assess the role of the negative entries within the adjacency matrix in the energy cost evaluation. Even when the system matrix is defined from sFC, with or without setting negative entries set to zero, the trends were sufficiently stable over time and above those obtained from dFC, independently of the control time. By contrast, at group level, the inter-individual NCT analysis showed a slightly higher variability (CoV ranging between 5% and 10%) than the intra-individual analysis, as also confirmed in the summary energy-related NCT feature introduced to directly index the dynamic energy efficiency of the human connectome. The higher inter-individual variability could reflect to some extent the individual specificity of the FC profiles (Cole et al., 2014; Finn et al., 2015; Mueller et al., 2013), which would plausibly affect the internal neural processes at the base of state transitions in terms of dynamic energy cost and efficiency. However, the energy-related feature showed a replicable inter-individual variability across different sensorineural conditions (NH vs. HL) and age-ranges (HCP vs. NH/HL) and did not acceptably discriminate between NH and HL.

Overall, intra- and inter-individual simulations confirmed that, when reducing the control times below a certain value (<50), the dynamic time-variant nature of the system appears consistently indexed by the emerging gap between Log-energy values for static and dynamic conditions. In contrast, for higher control times (>150), the gap between Log-energy values of static and dynamic conditions appears substantially reduced or absent. Independently from the sensorineural condition and the age range, this essentially confirmed the general advantage for the system (connectome), in terms of energy costs, of relying on a dynamic, rather than static, connectivity with adjacency matrix updated over time (Deng et al., 2022).

In line with the NCT literature (Deng et al., 2022; Gu et al., 2015; Karrer et al., 2020; Parkes et al., 2024), AC and MC of the system from large-scale brain networks were also included in the variability and discriminability analyses. We noted that the dynamic AC was on average higher than static AC, whereas the dynamic MC was on average lower than static MC for the three datasets. This result confirmed to some extent the energy-related results, suggesting that the intrinsic architectural advantage of the system when the adjacency matrix acquired the extra degree of freedom of being updated over time based on dynamic functional connectivity, could not only determine a relevant reduction of the energy consumption to achieve a specific state transition but also show a greater dynamic energy efficiency to switch across easy-to-reach state configurations.

The controllability features showed reduced inter-individual variability compared to energy-related features (CoVs between 1% and 5%), even if slightly higher values were obtained for the HCP dataset (CoVs ∼ 5%). In this case, however, a low inter-individual variability was not to be expected (or requested), and this could just reflect the presence of reliable FC patterns within homogeneous cohorts, confirming to some extent the good stability of NCT parameter estimates already observed in previous studies, across repeated scans (see, e.g., W. H. Lee et al., 2020) or for specific large-scale networks (R. Li, Yin, et al., 2017; Mueller et al., 2013) that included the ones considered here.

### Relevant Findings for NCT Application to Aging and HL

Overall, AC and MC mean values in the HCP cohorts were higher than the corresponding values from the other two cohorts (NH and NL), suggesting a plausible impact of the different age range on the estimation of these metrics. Controllability features also showed significant differences between the groups, for AC, in the DMN and VIS networks, and for MC, in the DMN, with an acceptable discriminability power (AUC > 0.7).

These results also confirmed to some extent the considerations presented in Deng et al. (2022), where NCT metrics were used to predict behavioral performance scores. Based on our results, NCT controllability features seems to have good potential for detecting a damage of the human connectome secondary to the worsening of peripheral sensorineural conditions, with a distinct neural plasticity response to aging. Indeed, the pathological condition of HL subjects is known to be characterized by a chronically reduced natural stimulation of the brain via the primary auditory pathway (Ponticorvo et al., 2019) and now, in light of these results, this seems to be associated with an altered functioning of the DMN, as indicated by the significant reduction of the AC, but also with higher reachability of certain nonconsolidated brain states (i.e., states that would be otherwise more difficult to reach). The significantly higher static and dynamic MC of HL, compared to NH, would therefore reflect the abnormally enhanced capacity or tendency of DMN nodes to drive the brain toward less consolidated activation states, a scenario that might interfere with normal cognitive functioning and predispose toward later neurological or neuropsychiatric disease. In addition, the sensorineural conditions positively affected the AC (most likely as a compensatory mechanism), although the poor discriminability would suggest limited applicability as a marker of brain plasticity. On the other hand, the clinical correlations of the static MC observed for the VIS network were significant only for the case of SC, suggesting that the VIS network would tend to increase its capability to drive the brain toward less-consolidated states but only proportionally to the plasticity of the underlying structural connections in association with the sensorineural deficit.

The complementarity of NCT and NGT modeling was already explored in Deng et al. (2022) where the combination of NCT and NGT features achieved better results than individual features in predicting behavioral scores. This was in part to be expected because NCT features are certainly affected by the graph topology (Cornblath et al., 2020), thereby NCT and NGT metrics could be correlated to some extent, especially because the estimation of the metrics was systematically performed on the same adjacency matrices (i.e., the same data). On the other hand, when analyzing the NCT and NGT metrics separately, NGT features showed significant differences in the VIS network (as already described in Ponticorvo et al., 2022), whereas NCT features indicated significant differences for the DMN network (not previously observed). This clearly suggests that NCT and NGT features can be sensitive to different aspects of neural processing, the former most likely capturing aspects of causality within the (functional) connectome that are not accounted for by the latter, and vice versa for alterations pertaining mainly to the topological properties (Srivastava et al., 2022). A few previous works (Bernhardt et al., 2019; Dimulescu et al., 2021; Wilmskoetter et al., 2022) have similarly compared NCT and NGT features, albeit deriving the respective model parameters only from the SC. More specifically, in Bernhardt et al. (2019), the two models gave similar results, supporting the idea that the topology constrains neural dynamics. In the other studies, however, the complementarity of these two methods emerged more clearly, with NTC being able to provide insights beyond the topological information contained in the NGT metrics. In fact, these analyses disclosed the idea that the structural connectome may indeed affect the brain dynamics, although the role of FC was not addressed. Thus, our findings based on both FC and SC data, not only corroborate the complementarity between NGT and NCT features but also unified the previous views on the problem, thereby advancing the interpretability of NCT features in a clinically relevant context.

### Limitations, Strengths, and Future Directions

Despite the above results, this study leaves some unresolved aspects. First, we derived NCT features using a cortical parcellation to link the interpretation of NCT results to the NGT results available from a previous report on the same datasets (Ponticorvo et al., 2022). Therefore, future work is needed to address the role of subcortical regions when these are added as further dimensions to the model and the impact of using a different parcellation. Second, a nontrivial, yet important, aspect concerns the normalization of the adjacency matrix in the implementation of the NCT analysis. In fact, especially the dynamic energy efficiency results could be strongly affected by the type of normalization employed for the evaluation of control energy features. Particularly, the stability of the system must be ensured. Here, we guaranteed the stabilization using two different approaches for the calculation of energy cost and controllability features. For energy cost estimation, we used the weighted Laplace matrix and exploited the Lyapunov equation for asymptotically stable systems as an alternative to using the integral form for the Gramian controllability (Poznyak, 2008). Conversely, in the controllability analysis, we switched from continuous- to discrete-time system modeling and chose an approach that was previously validated for discrete systems. This normalization scheme is less affected by the dimensionality (number of nodes) (Karrer et al., 2020) and ensures the Schur stability (i.e., restricts the eigenvalues within the unitary circle) but it is not yet clear how to best adapt this solution for the control energy simulations. Finally, while age-related HL is a highly prevalent pathology compromising the natural aging of the brain (Dalton et al., 2003), with a significant impact on daily life activities and quality of life, it is certainly not the only interesting case of sensorineural defect affecting the brain connectivity. For this reason, the variability of the NCT features was additionally verified on a different normative dataset, including younger adult subjects, albeit different effects could still be present in pathological subjects within the same age range. While several studies have shown neural mechanisms of degeneration in the pathophysiology of HL (Golub et al., 2017) (Lin & Albert, 2014), future studies on other neurologically impaired subjects could possibly further clarify how NCT modelling can be effective to describe neurodegeneration.

Despite these limitations, including a comparison between two alternative models (NCT, NGT) across two independent datasets has certainly strengthened this study. We explored the potential of using and interpreting metrics derived from NCT as biomarkers to describe the human connectome across different pathological conditions. In addition, the detailed analysis on the variability of NCT metrics represents a crucial phase because it validates their use in clinical studies. Thus, this work allows to move forward with the NCT application toward a new scenario where NCT features could be applied to investigate brain functioning under normal and disease-modified conditions or, eventually, to design novel paradigm for targeted neural stimulations (M. H. Lee et al., 2013), by incorporating suitable machine learning approaches to robustly predict conditions or treatment outcomes in new patients. Nonetheless, it will be also important to consider the use of other (more recent) NCT resources (see, e.g., Parkes et al., 2024) as well as to explore alternative approaches to define states (see, e.g., Ceballos et al., 2025; Cornblath et al., 2020).

## CONCLUSIONS

The present work demonstrates how NCT can provide multiple and valuable connectomic features from the suitable combination of structural and functional connectivity MRI data, enabling a more complete description of dynamic brain functioning under normative conditions and its alterations in the presence of aging-related sensorineural impairment. At the individual subject level, heuristic measures of dynamic energy efficiency can provide useful features provided that a proper matrix normalization approach is applied to ensure the stability of the linear system. At the population level, AC and MC exhibit even lower inter-individual variability across a wide range of (healthy) aging and promisingly acceptable discrimination performances with respect to a typical and highly prevalent aging-related condition that is well known to configure an important risk for cognitive impairment. Finally, combining NGT and NCT frameworks may provide complementary views and insights respectively into the spatial (topographical) and temporal (causal) organization of the human connectome, an aspect that will further encourage future studies on neural plasticity and connectivity effects in response to neurological disorders.

## SUPPORTING INFORMATION

Supporting information for this article is available at https://doi.org/10.1162/NETN.a.36.

## AUTHOR CONTRIBUTIONS

Simone Papallo: Conceptualization; Data curation; Formal analysis; Methodology; Visualization; Writing – original draft; Writing – review & editing. Alessandro Pasquale De Rosa: Data curation; Writing – review & editing. Sara Ponticorvo: Data curation; Writing – review & editing. Mario Cirillo: Data curation; Supervision; Writing – review & editing. Mario Sansone: Conceptualization; Writing – review & editing. Francesco Di Salle: Conceptualization; Data curation; Writing – review & editing. Francesco Amato: Conceptualization; Writing – review & editing. Fabrizio Esposito: Conceptualization; Data curation; Formal analysis; Funding acquisition; Investigation; Methodology; Project administration; Resources; Supervision; Writing – original draft; Writing – review & editing.

## FUNDING INFORMATION

Work supported by #NEXTGENERATIONEU (NGEU) and funded by the Ministry of University and Research (MUR), National Recovery and Resilience Plan (NRRP), project MNESYS (PE0000006) – A Multiscale integrated approach to the study of the nervous system in health and disease (DN. 1553 11.10.2022).

## DATA AVAILABILITY STATEMENT

All data that support the findings of this study are available upon reasonable request to the corresponding author.

## ETHICAL STATEMENT

The study is based on data from human experiments carried out in accordance with The Code of Ethics of the World Medical Association (Declaration of Helsinki) for experiments involving humans. The local ethical committee had originally approved the study and written informed consent had been signed by each participant before MRI acquisition.

## TECHNICAL TERMS

Connectome: Complete representation of all structural or functional connections among all brain regions from a predetermined brain parcellation.
Network control theory: Mathematical framework relating the topology of a network to the control of a dynamic input-state-output system.
Adjacency matrix: Square matrix expressing the first-order differential state equations of a linear dynamic system with an internal network structure.
Sliding time window: Moving time interval of fixed duration used for estimating functional connectivity between two brain regions.
Controllability: Capability of the system (brain) to efficiently complete transitions between reachable states via stimulation of specific nodes.
Control time horizon: Time unit for expressing the duration of brain transitions from one (initial) state to the next (or final) state.
Sensorineural hearing loss: Pathological condition of hearing loss, typically age-related and primarily affecting higher acoustic frequencies.
Input energy signal: Amount of energy injected into the nodes of the connectome potentially causing changes in the brain state across time.
Energy efficiency: Capability of the system (brain) to complete a transition between two states with reduced (or minimal) energy cost.
Brain state: Time-varying vector expressing the neural activity at all nodes (regions) of the connectome, quantifying the brain condition at each time point.
